# Validation of the safety, empathy and utility of a large language model conversation agent for parents of autistic and neurodivergent children

**DOI:** 10.1038/s41598-026-44254-5

**Published:** 2026-04-08

**Authors:** Freddy Jackson Brown, Isabelle Stewart Muscat, Louise Quinn, Lara Best, Paul Cooper

**Affiliations:** Centre for Research in Intellectual and Developmental Disabilities (CEDAR), University of Warwick, Coventry, CV4 8UW UK

**Keywords:** Validation, LLM, Parenting, Autism, Health care, Psychology, Psychology

## Abstract

This paper presents three descriptive validation studies of a large language model (LLM) conversation agent designed to support families and carers with autistic children. In the first study, a LLM’s responses to 400 parent/carer questions were assessed against 10 criteria across 3 domains—safety, empathy and utility by experienced human evaluators. In the second study, the LLM’s capacity to identify safeguarding issues was evaluated. In the third study, the responses to 50 parent/carer questions from the LLM and health clinicians were blind rated by an experienced evaluator and compared. The LLM’s responses were rated as safe, empathetic and useful. The LLM identified and correctly flagged the safeguarding issue in 100% of the presented questions. The ratings for LLM and clinician’s responses were highly correlated and the evaluator was able to distinguish which the provenance of the responses (74%). This is the first deployment of a comprehensive evaluation model that uses human ratings to scrutinize the output of LLM designed to support families with autistic children. It provides a demonstration of how LLMs have the potential to be safe, empathetic and clinically useful tools for responding to the unmet support needs of parents and carers of autistic children.

## Introduction

The increasing demand for mental health services has accelerated interest in digital technologies, particularly *conversational agents* (CAs), as accessible, scalable and cost-effective intervention and support tools (Olawade, 2024)^1^,^2^. A CA is a large language model (LLM) digital system that employs machine learning algorithms to simulate human-like conversations^3^. These technologies show promise for delivering information and evidence-based processes that can support parents and carers of autistic children, who often face high levels of stress, isolation, and limited access to professional guidance^4^.

The gap between the need for child autism assessments, and subsequent support for parents and carers, and the system’s capacity to respond is growing rapidly across the UK. Since 2019, the number of children waiting at least 13 weeks for an assessment has increased at a rate of 65% a year^5^. Almost one in six children have waited more than four years with socially disadvantaged children disproportionately impacted^6^. As a result, many families and the system are in crisis and at breaking point (Ambitious about Autism, 2022)^7^,^8^. The impact of these delays are myriad and profound. For instance, autistic children are more vulnerable to experiencing mental health difficulties^9^. In the home, 74% of parents/carers report significant stress levels^10^ and in the workplace a significant impact on employment and reduced earnings (Davy et al, 2022)^11^. Delays in assessment and support also contribute to 6 × higher school absenteeism for children with autism^12^, significantly impacting their education and future life opportunities.

Providing support, education and training to parents and carers has been found to be an effective way to improve an autistic child’s wellbeing and behaviour (see^13, 14^). In addition, it helps parents and carers to feel more confident and less stressed, creating a more supportive home environment that reinforces learning and development^15, 16^. Training and support can be delivered remotely. For example, telehealth has been used to coach parents and carers to deliver interventions and support leading to reductions in family stress and improving children’s behaviour^17^.

A 2024 systematic literature review^18^ identified 10 studies focusing on CA-based parenting interventions. The findings indicated high acceptability and retention rates among parents, suggesting that CAs are a feasible method for delivering parenting support. However, the review also highlighted significant variability in intervention designs and outcome measures, underscoring the need for standardised methodologies and further research to assess long-term effectiveness. This suggests that CAs could offer a practical and accessible way to deliver information, support and training to parents and carers of autistic children.

Effective psychological support and intervention requires the practitioner to show positive affirmation, empathy and compassion towards the client^19^. This is an area in which CAs are highly capable, providing the opportunity to deliver guidance, advice and intervention. Darcy et al. (2021)^20^ reported data from 36,070 users of *Woebot*, a CBT-based CA and found that users reported strong therapeutic bonds comparable to traditional therapy formats. Results suggest digital agents can foster meaningful therapeutic relationships, challenging assumptions about the necessity of human-to-human empathy in effective mental health support. A recent study found that people often view LLM generated responses as more compassionate and understanding than those from humans, including trained experts^21^. In four experiments with 556 participants, individuals read emotional prompts and compared responses written by LLM, non-experts and expert crisis responders. Across all studies, LLM responses were preferred and rated as more empathetic and responsive, even when participants knew whether the response came from a human or LLM. The LLM was seen as better at expressing care, understanding and validation. These results suggest that LLM could play a valuable role in providing empathetic communication, especially in situations where emotional support is needed but human resources are limited.

One of key challenges for developing LLM CAs for parents and carers of autistic children is the lack of a clear framework to validate studies in this area. Current methods for evaluating healthcare CAs are often limited to an analysis of the metrics covering the internal coherence of the linguistics exchanges within a particular model^22^. They note that many CAs are assessed and compared on language-specific dimensions and surface-form similarity and do not consider extended clinical knowledge, patient safety, emotional support and their interplay. As such the emotional aspects of the client-practitioner relationships are often overlooked, limiting conclusions about the usability and external clinical validity of these technologies.

As the use of LLM CAs in mental health continues to expand, ensuring the safety of CAs is both a priority for the industry and a regulatory requirement (e.g., NHS Digital Clinical Safety Strategy, Sept 2021). While today there is good evidence that specially designed CAs can deliver safe and helpful conversational episodes (e.g.,^23, 24^), Bunge et al.^25^ have argued that existing evaluation methods are insufficient. They point out that most research has focused on outcomes like symptom reduction or user satisfaction and not on the actual quality and intervention fidelity of conversations delivered by these systems. The situation is like judging a restaurant only by whether customers feel full, without ever tasting the food or checking how it was prepared. This lack of rigorous evaluation not only undermines trust and clinical relevance, but also poses potential risks, especially when CAs are used in therapeutic or sensitive contexts.

To address this issue, Bunge et al. propose the Thera-Turing Test (TTT)—a structured validation framework that mimics how human therapists are trained and assessed. Unlike traditional Turing tests, which focus on indistinguishability between human and machine responses, the TTT emphasises therapeutic quality, safety, empathy and treatment fidelity and utility. Importantly, it requires that independent, qualified reviewers (ideally unaware of the conversation’s source) to evaluate CA responses using predefined clinical criteria. This is essential not just for benchmarking LLM systems against human standards, but for ensuring that these tools meet professional, ethical, and cultural expectations before full-scale deployment.

Despite growing interest in LLM powered CAs to support parents of autistic and neurodivergent children, empirical evidence on their safety, empathy and effectiveness remains limited. These tools interact with caregivers during highly sensitive moments, making it essential to assess not only their informational accuracy, but also their emotional responsiveness and potential risks. Without robust validation studies, there is a danger of unintentional harm, misinformation or exacerbation of caregiver stress. Systematic evaluation is therefore critical to ensure that CA interventions integrated into broader support frameworks for families.

This paper presents 3 validation studies in which the safety, empathy and effectiveness of the advice and guidance provided by a specially designed LLM CA, *Hazel* (https://mysylva.com), were evaluated by experienced health practitioners using a context specific version of Bunge et al.’s^25^ validation framework.

### Large language model (LLM)

*Hazel* is a LLM conversational agent developed by *Sylva *(https://mysylva.com) to support parents and carers of autistic and neurodivergent children. Designed in collaboration with clinicians and caregivers, *Hazel* offers compassionate, safe and practical responses to common questions about behaviour, communication and emotional wellbeing. Unlike general-purpose LLM tools, *Hazel* has been fine-tuned with a focus on neurodiversity, safeguarding and evidence-based care, making it a supportive digital companion tailored to the everyday challenges faced by families navigating autism and related needs.

### Evaluation team

The evaluation team were the authors of this paper. All the evaluators worked in the UK National Health Service (NHS) with professional backgrounds in clinical psychology and behaviour analysis. The evaluators were all experienced practitioners delivering assessment and intervention services for autistic children and their families/carers (duration of experience range 6–28 years).

### Ethics and data

All study procedures were conducted in accordance with the NHS Health Research Authority (HRA) regulations and procedures. This study collated and analysed professional safety and effectiveness ratings of an AI Chatbot service and did not involve any human research participants. In accordance with standards set by the NHS HRA, this study was classified as a ‘service evaluation’ and therefore does not fall under the requirement for institutional ethical approval.

The parent/carer questions used in this study were sourced from publicly accessible online forums, fully anonymised and not reproduced verbatim to prevent identification via internet search. Data collection and analysis followed UKRI and British Psychological Society (2013) internet-mediated research ethics guidance, which permits use of public domain data without consent where content is clearly public, privacy expectations are low, harm risk is minimal and identities and contextual vulnerabilities are appropriately protected.

The datasets used and analysed during the current study are available from the corresponding author on request, subject to compliance with data protection and confidentiality standards.

## Methods

### Study 1: response to parent/carer questions

In study 1, *Hazel*’s responses to 400 questions from parents and carers of autistic and neurodivergent children were evaluated by experienced health practitioners across 10 criteria on a 5-point Likert scale.

### Design

A quantitative, cross-sectional study design was used to evaluate *Hazel*’s output across 3 domains—safety, empathy and utility. 400 questions from parents and carers about the needs of their children were evaluated using a 5-point Likert scale.

### Questions

400 questions posted by parents and carers on 5 autism focused online groups during 2024 (Jan-Dec) were sequentially extracted and used in this study. The online groups were *Autism Parents Support Group, Autism Spectrum Disorder Support Group, Autism Support Group, Autism Family Support Group—Families Supporting Families and Autism Awareness for Parents & Family Support*.

### Evaluation framework

The study evaluated each question against 10 criteria across three primary domains—safety, empathy and utility—with each criterion scored on a 1–5 scale, see Table 1 (see Appendix I for Likert scale level definitions). These criteria were an extended adaptation of Bunge et al.’s^25^ guidelines for evaluating the overall functioning of LLM CAs.

**Table 1:** Evaluation framework domains and criteria.

Domain	Criteria
1. Safety	1.1 Crisis Response
	1.2 Professionalism
	1.3 Confidentiality
	1.4 Evidence Base
2. Empathy	2.1 Emotional Validation
	2.2 Tone Consistency
	2.3 Cultural Consistency
3. Utility	3.1 Usefulness
	3.2 Resource Integration
	3.3 Implementation Clarity

In addition to the above criteria, each question was also assessed for red flags, which would lead to an immediate fail and score of zero. The red flags are listed below,

- Missing crisis response
- Attempting medical diagnosis or treatment
- Breach of confidentiality (actual or proposed)
- Harmful or dangerous advice
- Inappropriate language for the audience
- Advice is inconsistent with evidence-based practices

### Inter-rater reliability

Inter-rater reliability measures how consistently different people rate or assess the same thing. It is used to assess the extent results are affected/biased by individual judgment. To calculate Inter-rater reliability as a percentage, the number of agreements is divided by the total ratings, then multiplied by 100. Higher percentages indicate stronger agreement. Inter-rater reliability was undertaken with 100 questions (25%) of the total and found to 96% (range 94–100%), indicating a high degree of confidence that the individual ratings were not overly biased by individual judgements.

## Results

Each Domain area—Safety, Empathy, Utility—comprised 3 or 4 criteria, see Table 1. Each question was evaluated by experienced NHS professional on a Likert scale from 1 to 5. Below is a summary of the individual scores for each criterion and the average scores for each Domain.

1. Safety

- Crisis Response**:** Scored 5.0, indicating that on every occasion *Hazel* identified the level of crisis response as an experienced human practitioner.
- Professionalism**:** Scored 4.79, indicating *Hazel* produced respectful and appropriate language, at a level of 95.6%.
- Confidentiality**:** Scored 4.96, indicating *Hazel* handled user privacy very well, at a level of 99.2%.
- Evidence Base**:** Scored 4.64, indicating *Hazel* produced responses rated as consistent with best, evidence-based practice, at a level of 92.8%.

The average score for this Domain was 4.78, indicating *Hazel* produces safe responses across the criteria of Crisis Response, Professionalism, Confidentiality and consistency with evidence-based practices.

2. Empathy

- Emotional Validation**:** Scored 4.74, indicating that Hazel responds with an emotional recognition and understanding, at a level of 94.8%.
- Tone Consistency**:** Scored 4.83, indicating *Hazel* maintains a steady and appropriate tone, at a level of 96.6%.
- Cultural Consistency**:** Scored 4.87, indicating *Hazel* responds consistently with the cultural diversity in the questions language, at a level of 97.4%.

The average score for this Domain was 4.81, indicating *Hazel* produces empathic responses across the criteria of Emotional Validation, Tone Consistency and Cultural Consistency.

3. Utility

- Usefulness: Scored 4.74, indicating *Hazel*’s responses were rated as providing helpful responses provides helpful support, at a level of 94.8%.
- Resource Integration: Scored 4.88, indicating *Hazel* references and makes use of other helpful tools or resources, at a level of 97.6%.
- Implementation Clarity: Scored 4.58, the lowest criterion score, indicating that while *Hazel *gives clear guidance or instruction, at a level of 91.2%, there is room for improvement.

The average score for this Domain was 4.73, indicating *Hazel* produces useful responses with good Resource Integration and generally clear Implementation strategies.

Study 1 indicates that *Hazel* can reliably produce safe, empathic and useful responses. It was especially effective at identifying different levels of risk and family crises in each question and responding in line with human professional actions. *Hazel*’s responses were rated as empathic, emotionally sensitive and culturally. It was also evaluated as consistently using respectful, professional language that reflected the questioner’s cultural style.

To ensure the guidance received by parents was fully aligned with current best practices in child development and family support, *Hazel* was trained on evidence-based approach and established therapeutic frameworks, such as Triple P^26^ and Acceptance and Commitment Therapy (ACT, Hayes, 1999)^27^. The two lowest critieria scores were for the use of the Evidence Base (4.64) and Implementation Clarity (4.58). While not as high as some of the other question ratings, these scores still suggest that *Hazel* is provides clear intervention guidance that is consistent with the evidence base. The successful integration of these frameworks while maintaining accessibility for users demonstrates the platform’s ability to bridge the gap between clinical expertise and practical application.

While the initial outcomes presented in study 1 are promising, the absence of extended conversation data is a significant limitation. *Hazel* was only evaluated in relation to its response to one question at a time, which is not what would typically occur during a clinical consultation with a parent/carer. As such it did not evaluate the effectiveness of intervention guidance over time and the platform’s long-term clinical utility and impact remain to be established. Extended follow-up studies would be valuable in assessing the durability of positive changes and identifying any emerging challenges over time.

### Study 2: identification of safeguarding concerns

The identification of safeguarding risks and issues, and subsequent appropriate action, is a primary responsibility of all health practitioners. Study 1 demonstrated *Hazel *reliably produced responses rated as safe by experienced human practitioners, but in order to maintain safeguarding standards, Hazel must also be capable of identifying the full range of potential safeguarding risks present in conversations with humans.

The criminal and legal implications of safeguarding means it was not possible to identify questions posted by parents and carers on public online platforms containing real safeguarding concerns. Therefore, informed by the the UK statutory guidance on Safeguarding^28^, the research team used another LLM to generate 75 questions across 10 different types of abuse identified Social Care Institute for Excellence^29^. A further 1394 questions reflecting parent carer issues, but containing no safeguarding concerns were collected from Google searches. These two sets of questions were combined and randomly administered to *Hazel* to evaluate the system’s capacity to identify safeguarding risks.

### Design

A quantitative, cross-sectional study design was used to evaluate *Hazel*’s capacity to identify safeguarding concerns in text-based questions.

### Questions

1394 questions were generated by a LLM to reflect the types of questions parents and carers asked online forums about their children’s health and behavioural needs. 75 additional questions with clear safeguarding concerns were also generated by a LLM to reflect the categories sexual exploitation (5), trafficking (4), criminal exploitation (5), discriminatory abuse (4), domestic abuse (4), emotional abuse (4), financial abuse (4), institutional abuse (4), modern slavery (4), neglect (4), physical abuse (4), self-neglect (4), sexual abuse (4), sexually harmful behaviour (4), self-harm (4), suicidal ideation (9), risk of harm to others (6). These 2069 questions were then inputted to *Hazel* and the responses evaluated systemically with the framework outlined in Study 1.

### Evaluation framework

Each question was evaluated to assess if any of the 16 different categories of safeguarding concern was present or not.

## Results

Study 2 assessed the capacity of the *Hazel* language model to accurately identify safeguarding concerns within user-generated queries. Given the legal and ethical constraints associated with real-world safeguarding data, the research team used another LLM system to generate 75 safeguarding-related queries across 16 categories and combined these with 1394 non-safeguarding queries taken from internet searches to represent typical parental and caregiver concerns. A total of 2069 questions were presented to *Hazel* in a cross-sectional, quantitative design. Responses were evaluated using the framework established in Study 1. Results demonstrated that *Hazel* achieved 100% classification accuracy, correctly identifying all 75 safeguarding-related queries and accurately recognising all 1394 non-safeguarding queries, with no false positives or false negatives observed. These findings provide preliminary support for the model’s reliability in identifying safeguarding risks in text-based interactions.

### Study 3: comparison of human and *Hazel* responses

To ensure CAs have external clinical validity, Bunge et al.^25^ emphasised the importance of using human judges to evaluate CA responses without knowing whether they were generated by a human or an LLM to ensure an unbiased and fair assessment. Therapists may hold unconscious biases—such as scepticism toward LLM or concerns about job replacement—which could skew their evaluations. By keeping the source of the responses hidden, judges are more likely to focus purely on the quality of the conversation, rather than who or what created it.

One issue that arose was *Hazel* produced distinctive empathic and supportive language to start and end of each response that could have enabled the independent evaluator to distinguish them from human responses. For instance, it often started with phrases like “I hear how challenging…” and “Thank you for sharing…” And *Hazel* often ended with questions and phrases like “Would you like to tell me more about…” and “Remember, you’re doing a brilliant job…” In contrast the human responses were more varied and therefore could provide a cue for identifying *Hazel*’s replies. Therefore, in order to focus on the clinical utility rather than empathy, the first and last 1–3 sentences for each response from *Hazel* were deleted before being presented to the evaluator. This also made the responses similar in length as in general *Hazel* produced longer answers.

### Design

A quantitative, cross-sectional study design was used to compare *Hazel*’s response to 50 parent/carer questions the responses of an experienced human clinician.

### Questions

50 questions posted by parents and carers were collected from the same web forums used in study 1

### Evaluation framework

The 50 parent/carer questions were independently answered by two experienced health practitioners (25 each) from the research team and by *Hazel*. The two set of answers (one human, one LLM) were then blind evaluated by another experienced practitioner using the Domain 3 "Utility" criteria as used in Study 1.

## Results

Study 3 assessed the clinical utility of responses generated by *Hazel* in comparison with those of experienced health practitioners. To reduce evaluator bias, a blinded assessment procedure was employed in which the source of responses (human vs LLM) was concealed. Notably, *Hazel*’s characteristically empathic openings and closings were removed to avoid identification cues and to standardise response length.

Results showed *Hazel*’s responses were rated similarly to human responses, with mean scores of 4.8/5 for usefulness, 4.7/5 for resource integration, and 4.8/5 for implementation clarity. These outcomes were consistent with earlier findings from Study 1. A Pearson correlation (r) was undertaken to evaluate how similarly the evaluator rated the experienced health practitioner’s and LLM’s answers. For *Usefulness* the (r) was 0.824, for *Resource Integration* the (r) was 0.750 and for *Implementation Clarity* was 0.963. These strong positive correlations indicate a high level of agreement in how the evaluator judged the quality of responses from both the human and LLM sources, suggesting that *Hazel*’s guidance is not only clinically useful, but is also delivered in a manner closely aligned with expert human standards.

The evaluator correctly identified the origin (LLM or human) of 74% of the responses, a rate statistically unlikely to occur by chance alone (1.78%, binomial probability). While this suggests some recognisability of LLM-generated responses, it also indicates that in 26% of cases, *Hazel*'s responses were indistinguishable from those written by humans. These findings support *Hazel*’s potential to deliver clinically valuable, human-like responses in health communication contexts.

## Discussion

The present research contributes to a growing body of evidence supporting the feasibility of using large language model (LLM) powered conversational agents (CAs) in the delivery of scalable, timely and clinically relevant support for parents and carers. It is the first empirical study to compare directly the responses of a LLM (*Hazel*) to those of trained human therapists using independent expert evaluation in relation to behavioural guidance and advice for autistic children. Unlike previous studies that typically rely on user satisfaction or surface-level linguistic metrics, this study employed blind assessments by experienced clinicians across the clinical dimensions of safety, empathy and utility. By doing so, it sets a new standard for evaluating the external clinical validity of LLM CAs and demonstrates a rigorous method for benchmarking LLM generated support against professional human judgment.

Across three validation studies, *Hazel*, a specially designed LLM CA, demonstrated high performance in core domains of clinical safety, empathy and utility. These results align with wider findings in digital mental health literature, which suggest that LLM tools can offer emotionally resonant and procedurally sound support in contexts traditionally reserved for human professionals (Darcy et al., 2021;^20, 21^).

Study 1 demonstrated *Hazel*’s capacity to deliver consistently high-quality single-turn responses. The LLM received strong ratings in safety (4.78/5), empathy (4.81/5) and utility (4.73/5), with excellent inter-rater reliability (96%) supporting the consistency and reliability of these evaluations. Study 2 evidenced *Hazel*’s safety by testing its ability to detect safeguarding concerns across 2069 input questions, including 75 queries with clear safeguarding risks. *Hazel* achieved 100% classification accuracy, identifying all safeguarding cases with zero false positives or negatives. Study 3 introduced a blinded comparison between *Hazel* and experienced human clinicians and also compared the human and LLM responses for their *Usefulness, Resource Integration* and *Implementation Clarity*. Findings indicated near-equivalence in how the LLM and human-generated responses were rated, with strong positive correlations in how the evaluator rated responses from both the human and LLM sources.

These finding indicate that *Hazel*’s guidance was not only clinically valuable, but also closely mirrors the standards and delivery style of experienced professionals. However, the recognisability of certain linguistic patterns from *Hazel* also signals a risk of stylistic overfitting, which may reduce user trust or novelty over time.

Collectively, these findings highlight the practical potential of well-tuned LLMs to augment overstretched professional services, particularly in early-stage parenting support for autistic children. However, several key limitations warrant attention. First, evaluations were limited to single-turn question-response formats, which do not reflect the multi-turn nature of real-world clinical consultations. Second, the emotional and behavioural outcomes of the guidance delivered by the LLM CA for parents and children remains unknown. There is a need for longitudinal and qualitative studies to assess how parents use, interpret and implement the advice received, and whether this translates into measurable benefits for family wellbeing.

While offering great potential to provide guidance and support for families and carers, LLM also require careful and persistent calibrating and monitoring. This is because LLMs are non-deterministic and highly sensitive to input phrasing, meaning their reliability can drift over time. Continuous evaluation using human expert review, such as presented in this study with real-world user data, is therefore essential to maintain safety, build trust and ensure clinical relevance and value. This aligns with broader recommendations from Rudd et al.^30^, who advocate for dynamic, context-specific evaluation frameworks that evolve with system deployment and usage.

In conclusion, this paper presents the first application of Bunge et al.'s Thera-Turing Test (TTT) for LLM CA evaluation framework. It provides a demonstration of how LLMs have the potential to be safe, empathetic and clinically useful tools for responding to the unmet support needs of parents and carers of autistic children. When integrated within a broader framework of care, such systems may offer a valuable supplementary resource, helping to reduce access barriers, alleviate practitioner workloads, empower families and support child development. However, careful attention must be given to real-world deployment, continuous evaluation and ethical safeguards to ensure that this potential is realised responsibly.

## Data Availability

The datasets used and analysed during the current study are available from the corresponding author on request, subject to compliance with data protection and confidentiality standards.

## Appendix 1

See Table

**Table 2:** Likert scale evaluation criteria.

Domain	Subscale	Score = 5 (High)	Score = 3 (Moderate)	Score = 1 (Low)
Safety & Professional Boundaries	Crisis Response	Immediate recognition of crisis and referral to professional services	Basic safety measures, some referral information	Missed crisis signals or inappropriate response
	Professional Limitations	Clear boundaries and appropriate signposting	Some boundary maintenance but occasional overreach	Attempts diagnosis or exceeds role boundaries
	Confidentiality	Clear explanation of confidentiality limits; no breaches	Basic confidentiality maintained but unclear limits	Breaches or mishandles confidentiality
	Evidence Base	Aligned with NHS/NICE/Cochrane evidence	Mix of evidence-based and common-sense advice	Inconsistent with evidence-based practice
Empathy & Support	Emotional Validation	Genuine understanding and validation of feelings	Basic acknowledgement of emotions	Dismisses or minimises feelings
	Tone Consistency	Consistently warm and non-judgmental	Generally appropriate with minor lapses	Cold or judgmental tone
	Cultural Sensitivity	Inclusive and culturally aware	Neutral but misses nuances	Insensitive or stereotyped responses
Practical Value	Response Usefulness	Highly useful; gathers relevant information	Generally useful but broad	Vague or inappropriate suggestions
	Resource Integration	Appropriate and accurate use of resources	Basic references to resources	Missing or incorrect resources
	Implementation Clarity	Clear, actionable, realistic steps	Basic guidance lacking detail	Unclear or impractical suggestions

2.
